# Photosensitizers in Motion: Micro- and Nanomotor-Assisted Photodynamic Therapy

**DOI:** 10.1021/acsnanomed.6c00135

**Published:** 2026-09-07

**Authors:** Flávia Amanda Pedroso de Morais, Katherine Villa

**Affiliations:** † Institute of Chemical Research of Catalonia (ICIQ), The Barcelona Institute of Science and Technology (BIST), Tarragona E-43007, Spain; ‡ Institució Catalana de Recerca i Estudis Avançats (ICREA), 08010 Barcelona, Spain

**Keywords:** micro- and nanomotors, photodynamic therapy, photosensitizers, active transport, self-propulsion, reactive oxygen species, hypoxia, antimicrobial therapy

## Abstract

Photodynamic therapy (PDT) is a clinically relevant and minimally invasive strategy for treating diseases associated with uncontrolled tissue growth, including nonmelanoma skin cancers and microbial infections. Its spatiotemporal selectivity minimizes damage to healthy tissues and limits the emergence of therapeutic resistance, while its versatility enables integration with complementary therapeutic approaches. One emerging direction is the combination of PDT with artificial self-propelled micro- and nanomotors (MNMs). Active MNM motion enhances photosensitizer (PS) transport and localization, while oxygen-generating or oxygen-supplying MNM platforms can partially mitigate hypoxia-related limitations of PDT. This Review provides a comprehensive overview of recent advances in MNM-assisted PDT, highlighting commonly used PS molecules and strategies for their integration into MNMs to couple photodynamic activity with autonomous propulsion. It examines representative *in vitro* and *in vivo* studies, identifies critical performance parameters and current challenges, and explores future opportunities for the rational design of next-generation self-propelled phototherapeutic systems.

## Graphical abstract

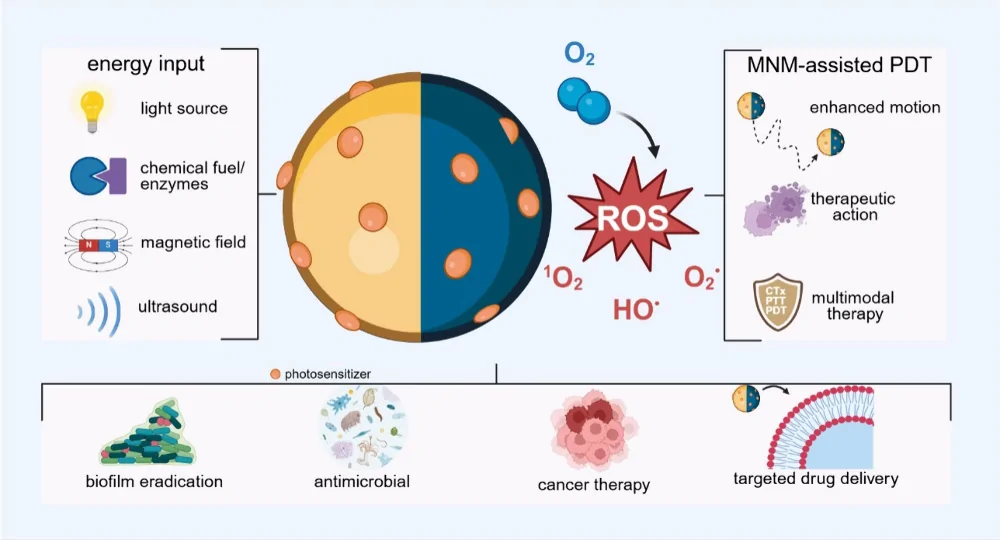

Light-based therapies have a long and distinguished history, dating back to ancient civilizations that used sunlight and natural compounds to treat infections, psoriasis, rickets, vitiligo, and other health conditions.^1,2^ However, it was not until the early twentieth century that these empirical practices evolved into a scientifically grounded therapeutic approach.^3,4^ This progression began with the pivotal discovery that specific combinations of light and chemical agents could induce cytotoxic effects, laying the foundation for phototherapeutic techniques.^3–5^

Currently, photodynamic therapy (PDT) is recognized as a highly effective, noninvasive therapeutic treatment of many microbial infections and other local pathologies. By inducing localized and targeted oxidative stress, PDT achieves selective damage to diseased or infected cells while minimizing the risk of drug resistance development.^3–5^ Despite these advantages, the broader clinical translation of PDT remains limited by intrinsic biological and physicochemical constraints, including hypoxia within diseased tissues, restricted penetration of therapeutic agents, and the short diffusion range and lifetime of the photogenerated reactive oxygen species (ROS).^6,7^

Conventional PDT formulations rely on passive diffusion of photosensitizers (PSs) to the target site, a process that is particularly inefficient in complex microenvironments such as solid tumors and biofilms.^7–9^ These limitations have motivated the development of strategies that incorporate active transport to locally enhance the photodynamic activity. In this context, micro- and nanomotors (MNMs) have emerged as a promising strategy to overcome transport and hypoxia-related limitations of conventional PDT through autonomous self-propulsion and multifunctional design.^6–8^ MNMs are active particles that generate motion by converting chemical energy or externally supplied energy, such as light, magnetic fields or ultrasound, or by exploiting the intrinsic motility of biological components in biohybrid systems . Importantly, they should be distinguished from passive nanoparticles that may be externally guided (e.g., magnetic steering) or functionalized for active targeting but do not themselves generate locomotion. Beyond enhancing transport, MNMs provide versatile platforms in which PS can contribute not only to ROS generation but also to structural design, catalytic activity, and motor functionality.^10–12^ Such integration enables the development of multifunctional theranostic systems that combine imaging, oxygen modulation, and synergistic therapeutic modalities within a single micromotor architecture.^13–17^

Considering that both MNMs and PDT fields rapidly evolve, a comprehensive understanding of their underlying mechanisms, synergistic interactions, and remaining challenges is essential for the rational development of integrated therapeutic platforms. This review summarizes recent progress in MNM-assisted PDT, and examines how micromotor-based approaches can address limitations of conventional PDT formulations, including insufficient target delivery and in selected oxygen-generating platforms, tumor hypoxia. A literature search was conducted using Web of Science, Scopus, and PubMed for studies published from 2015 to July 2026, using combinations of the terms “photodynamic therapy”, or “PDT” with “micro- and nanomotors”, “microswimmers”, or “nanorobots”. We first introduce the fundamental principles of PDTand discuss the main limitations that hinder its clinical efficacy. We then assess how MNMs can address some of these challenges by improving the transport, localization, and activation of PS within complex biological environments. Building on this foundation, we discuss the principal design strategies for MNM-assisted PDT, with emphasis on PS selection, integration methods, and the relationship between photophysical performance and dynamic behavior. Finally, we review recent *in vitro* and *in vivo* studies, identify the main challenges related to biocompatibility, controllability, and clinical translation, and outline future directions for the rational development of motion-enabled photodynamic therapies.

## General Mechanism of Photodynamic Therapy

The combination of molecular oxygen (^3^O_2_), a PS, and light of an appropriate wavelength is required to produce the photodynamic effect.^18^ Upon irradiation, the PS undergoes a transition to a short-lived excited singlet state (^1^PS*). In this state, ^1^PS* can dissipate its excess energy through non-radiative (e.g., vibrational relaxation, internal conversion) and/or radiative relaxation (e.g., fluorescence emission), returning to the electronic ground state (^1^PS), typically in its lowest vibrational level. Alternatively, ^1^PS* can undergo intersystem crossing (ISC) to form the longer-lived excited triplet state (^3^PS*). This triplet state shares a similar multiplicity with molecular oxygen (^3^O_2_), enabling energy transfer between them. Consequently, the ^3^PS* can undergo electron-transfer or hydrogen-abstraction reactions with nearby biomolecules or molecular oxygen (Type I), leading to the formation of radical intermediates and ROS such as hydrogen peroxide (H_2_O_2_), superoxide (O_2_^•‑^), and hydroxyl radicals (HO^•^). Alternatively, ^3^PS* can transfer its energy directly to molecular oxygen (Type II), producing ^1^O_2_ (Figure 1). These reactive species induce oxidative damage to cellular components, including lipids, proteins, and nucleic acids, ultimately leading to cell death. Therefore, the efficacy and selectivity of PDT depend on the efficient generation of ROS within the target tissue. This process is strongly influenced by the photophysical properties and localization of the PS, the characteristics of the light source, and the local oxygen concentration, which is often limited under hypoxic conditions.^19,20^

**Figure 1:**
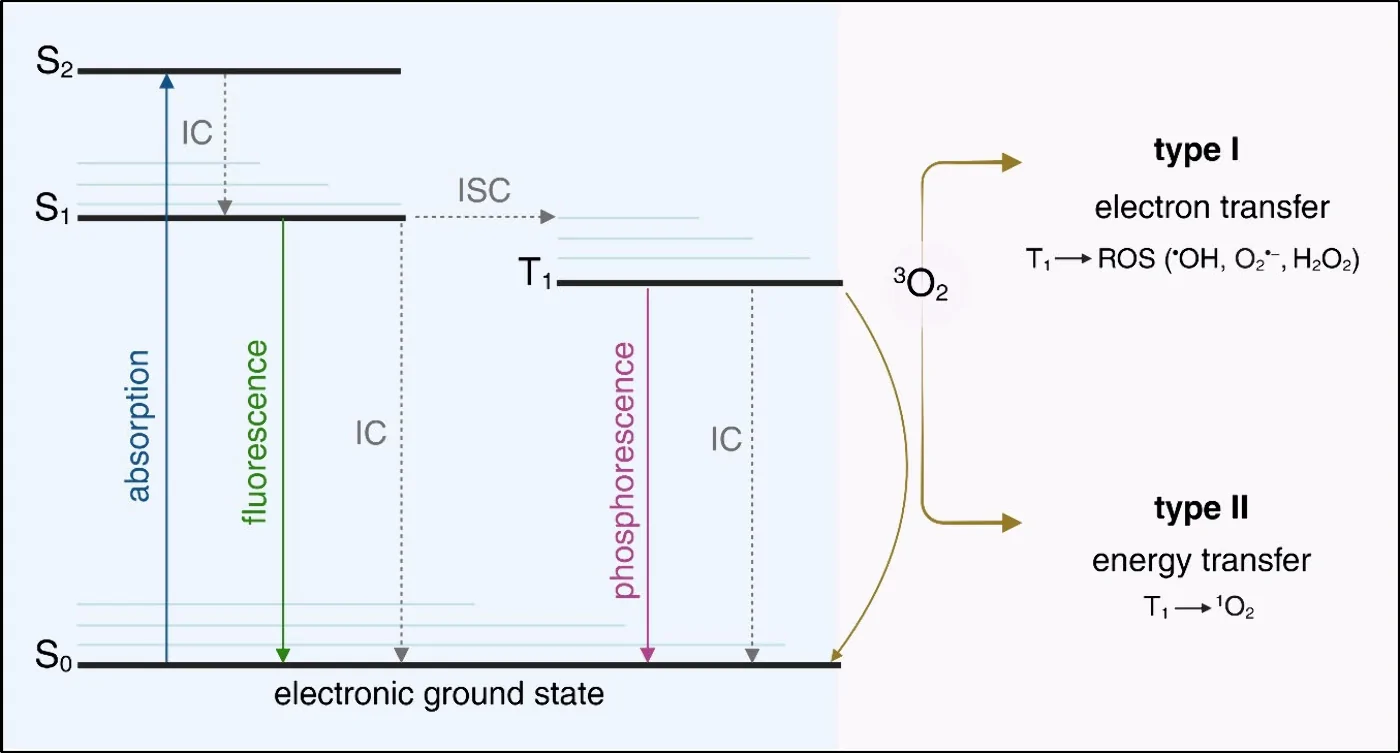
Photophysical and photochemical pathways in photodynamic therapy. Jablonski diagram showing photosensitizer excitation and relaxation, followed by Type I ROS generation or Type II singlet-oxygen production. Created in BioRender. Villa, K. (2026) https://BioRender.com/k48wvhu.

Beyond fundamental photochemical pathways, several intrinsic properties of the PS determine ROS generation and therapeutic performance, including photostability, aggregation behavior, triplet-state lifetime, and singlet oxygen quantum yield (Φ_Δ_). PS aggregation in biological media frequently leads to aggregation-caused quenching (ACQ), reducing fluorescence and excited-state lifetimes and, consequently, limiting ROS production. Thus, maintaining PS dispersed or using aggregation-tolerant materials is important for optimal PDT performance and provides an additional rationale for its integration with MNM. Photostability is also a critical factor, as photobleaching during irradiation can progressively reduce ROS generation, especially in prolonged treatments. Additionally, the triplet-state lifetime determines the time available for interaction with molecular oxygen or nearby biomolecules, whereas Φ_Δ_ reflects how efficiently absorbed light is converted into cytotoxic ^1^O_2_. While high Φ_Δ_ values are preferred for Type II PDT, their effectiveness remains dependent on sufficient local oxygen. Consequently, the ideal PS should combine robust physicochemical stability, long triplet-state lifetime, high ROS generation, and strong resistance to photobleaching.

Nevertheless, conventional PDT relies on PS compounds that mostly generate ^1^O_2_ via a Type II mechanism (energy-transfer), a process highly oxygen-dependent and therefore less effective under hypoxic conditions.^21–23^ Conversely, Type I pathways may retain activity under reduced oxygen concentrations because electron- or hydrogen-transfer reactions can generate radical intermediates. However, the subsequent formation of oxygen-derived ROS, including H_2_O_2_, O_2_^•‑^, and ^•^OH, still depends to some extent on molecular oxygen availability.

Catalytic strategies can further improve the PDT under hypoxic conditions through distinct mechanisms. Fenton or Fenton-like reactions convert endogenous H_2_O_2_ into highly reactive hydroxyl radicals in the presence of transition-metal ions, increasing oxidative stress without directly producing molecular oxygen. In contrast, catalase-like decomposition of H_2_O_2_ generates O_2_, which can partially alleviate local hypoxia and support oxygen-dependent type II photosensitization.^22,24^ Accordingly, recent developments have focused on Type I PSs, improved ROS generation under hypoxic conditions, and oxygen-supplying strategies.^25–27^

Overall, these developments have expanded the capabilities of PDT by improving the ROS generation and PS performance. Nevertheless, effective therapeutic outcomes ultimately depend not only on the properties of the PS but also on its delivery, localization, and activation within complex biological environments.^28–30^

## From Static to Dynamic PDT Formulations

Although considerable effort has been devoted to improving PS chemistry, the therapeutic performance of PDT is ultimately determined by both the properties of the PS and its transport within the biological environment. This has motivated the integration of MNMs, which introduce active transport into PDT and expand the functional role of the PS beyond ROS generation alone.^28–34^

Some PSs can actively modulate the propulsion behavior of MNM-assisted PDT, creating a bidirectional interplay between MNM motion and PDT efficacy.^35–42^ Therefore, PS-loaded MNMs can be broadly classified into two categories: (i) systems in which the PS primarily serves as a therapeutic payload, with active propulsion enhancing PS delivery, localization, and tissue accumulation and (ii) systems in which the PS acts as a propulsion-regulating component, enabling photoinduced processes to directly drive or modulate MNM motion.

Most reported MNM-assisted PDT systems belong to the first category, where the principal purpose of the MNM is to overcome the mass-transport limitations of traditional passive formulations and improve PS delivery to the target site. In these systems, active propulsion may enhance PDT efficacy via several complementary mechanisms including improved PS transport, intratumoral penetration, and more uniform distribution. Many of these systems further incorporate oxygen-generating catalysts (e.g., enzymes, Pt, or MnO_2_), oxygen carriers (e.g., proteins), or photosynthetic microorganisms (e.g., algae) to compensate for local oxygen depletion and improve PDT efficacy.^39,41^ In contrast, considerably fewer systems exploit the PS itself as a propulsion-regulating component. Representative examples include Janus IR820@CuS/Pt,^43^ walnut-shaped PDA–PEG-ICG-Fe^3+^,^44^ MPDA@ICG@Ur@Man,^45^ APIJNS,^46^ mPL-Pc@PDA,^47^ Au@ZnO@SiO_2_-ICG,^48^ and DIMs.^49^ Notably, except for mPL-Pc@PDA, nearly all currently described propulsion-regulating systems rely on near-infrared (NIR)-responsive PS, primarily indocyanine green (ICG) or structurally related cyanine dyes (e.g., IR820). These PSs are capable of efficient photothermal conversion under NIR irradiation, generating localized thermal gradients that simultaneously contribute to light-induced propulsion and enable PDT activation.

Although NIR irradiation offers greater tissue penetration than visible light and is therefore attractive for MNM-assisted PDT, its penetration depth remains limited in deep tissues. Furthermore, photothermal heating should be carefully controlled to avoid nonspecific thermal damage while preserving the therapeutic selectivity. This dual functionality exemplifies how strategic PS selection can directly shape both the therapeutic efficacy and MNM motility.

It is important to interpret the therapeutic benefits attributed specifically to MNM motion with caution as many reported platforms incorporate additional functionalities such as oxygen generation, photothermal conversion, chemotherapy delivery, or active targeting. As a result, observed improvements in PDT efficacy cannot always be attributed solely to propulsion, and the contribution of MNM motion should be evaluated within the context of each system design by using appropriate controls. Although some studies include component-specific comparisons, systematic evaluations against compositionally matched nonmotile counterparts remain limited, making it difficult to isolate the specific effect of active motion.

To date, MNM-assisted PDT has been investigated primarily for cancer therapy and the treatment of antimicrobial-resistant (AMR) infections.^50–52^ These applications have led to a broad range of MNM designs that differ in their PS integration, propulsion strategy, and therapeutic function. The following sections examine these design principles and discuss how they influence the performance of the MNM-assisted PDT platforms.

## Design Principles of Micro- and Nanomotor-Assisted Photodynamic Therapy

The rational design of MNM-assisted PDT systems requires the integration of propulsion, PS functionality, and biological compatibility within a single coordinated platform. Unlike conventional nanocarriers, MNMs must effectively address transport, photochemical, and therapeutic challenges simultaneously, making their design inherently multidimensional and complex. As a result, the selection of PS, propulsion mechanism, and the specific coupling strategy between the MNM and PS are pivotal in dictating both the overall efficacy of PDT and the potential breadth of biomedical applications. Table 1 summarizes the diverse PS that have been integrated into MNM platforms, along with their corresponding motor architecture, driving sources, and propulsion mechanisms.

**Table 1:** Overview of PSs Integrated into MNMs^a^

PS	MNM Architecture	MNM Properties	Energy Source	Propulsion Mechanism	Ref.
Porphyrins (e.g., TAPP)	Enzyme-based hollow mSiO_2_ (MHSTU)	s: 2 μm;	Urea (25 mM)	Self-diffusiophoresis	^53^
		v: ≈5.2 μm s^–1^			
		b: *E. coli*;			
		DL%/EE%: N/R			
	Enzyme-based MOF/UCNP (UTZCG)	s: 41 nm	H_2_O_2_ (50 mM)	Self-diffusiophoresis	^54^
		d: 0.8 μm^2^ s^–1^			
		b: HeLa cells			
		DL%/EE%: N/R			
	Enzymatic-based mSiO_2_, Au-coating and UCNP (UMSTCA3)	s: ≈50 nm	H_2_O_2_ (endogenous/50 mM)	Self-diffusiophoresis	^55^
		d: 1.7 μm^2^ s^–1^			
		b: 4T1, HepG2, HeLa, and B16 cells			
		DL%/EE%: N/R			
	Polymeric-based (PCM)	s: ≈150 nm	Light	Self- diffusiophoresis+ Self-thermophoresis	^42^
		v: 7.8 μm s^–1^			
		b: HepG2 cells			
		EE%: 84%			
					
Cyanine (e.g., ICG and IR820)	RBC-based IONPs (RBCM)	s: 2 μm	Ultrasound field	Acoustic propulsion	^56^
		v: ≈20 μm s^–1^			
		b: L02 cells, J774 macrophages, and RBCs			
		DL%: 0.2%			
	MOF-based (MIL88ICG@ZIF8DOX)	s: ≈100 nm	H_2_O_2_ (endogenous)	N/R	^57^
		v: N/R			
		b: 4T1 cells			
		DL%/EE%: 3.6%/89%			
	Matchstick-shaped Au@ZnO@SiO_2_-ICG Janus	s: ≈100 nm	NIR light	Self-thermophoresis	^48^
		v: N/R			
		b: *E. coli/S. aureus*			
		DL%/EE%: N/R			
	Mesoporous silicon-coated with peptides (DIMs)	s: ≈190 nm	NIR light	Self-thermophoresis	^49^
		v: 16 μm s^–1^			
		b: 4T1 cell			
		DL%/EE%: 4.2%/41.2%			
	HSA-modified with Au nanorods and FA (HANM@FI)	s: 275 nm	Urea (100 mM)	Self-diffusiophoresis + Self-thermophoresis	^58^
		d: 1.24 μm^2^ s^–1^			
		b: HepG2 and HL7702 cells			
		EE%: 47.7%			
	Polymeric-based (PDA–PEG-ICG-Fe^3+^)	s: 374 nm	NIR light	Self-thermophoresis	^44^
		v: 3.5 μm s^–1^			
		b: MCF-7 cell			
		DL%/EE%: N/R			
	l-arginine loaded into microneedles patch (PMV/ICG/l-Arg)	s: 300–400 nm	NIR light	Self-thermophoresis + gas propulsion	^59^
		v: 5.6 μm s^–1^			
		b: *S. aureus*			
		DL%/EE%: N/R			
	CuS sputtered with Pt (Janus IR820@CuS/Pt)	s: 142 nm	NIR light and H_2_O_2_ (endogenous or 10 mM)	Self-diffusiophoresis+ Self-thermophoresis	^43^
		v: 17.4 μm s^–1^			
		b: 4T1 and NIH-3T3 cells			
		DL%/EE%: N/R			
	Enzyme-based PDA (MPDA@ICG@Ur@Man)	s: ≈350 nm	Urea (100 mM)	Self-diffusiophoresis+ Self-thermophoresis	^45^
		d: 1.51 μm^2^ s^–1^			
		b: *E. coli/S. aureus*			
		DL%: 22.6%			
	Au_2_Pt and PMO-based (APIJNS)	s: 214 nm	NIR light and H_2_O_2_ (200 mM)	Self-diffusiophoresis + Self-thermophoresis	^46^
		v: 120.6 μm s^–1^			
		b: HeLa cells			
		EE%: 40.5%			
	Enzymatic-based C/SiO_2_ (CSIL)	s: ≈250 nm	NIR light	Self-thermophoresis	^60^
		v: 2.67 μm s^–1^			
		b: MRSA biofilm			
		EE%: 82%			
	Mesoporous Pt-organosilica (PPIC)	s: 276 nm	H_2_O_2_ (20 mM)	Bubble propulsion	^61^
		v: 3.32 μm s^–1^			
		b: 4T1, MCF-7 cells			
		DL%: 1.28%			
					
Chlorins (e.g., Ce6)	Enzymatic-based ZIF (GC6@cPt ZIF)	s: 122 nm	Glucose (10 mM)	Self-diffusiophoresis	^15^
		v: 2.08 μm s^–1^			
		b: 4T1 cells			
		DL%: 1.3%			
	ZIF-based with arginine-terminal (C&D@Z)	s: 83.97 nm	NIR light	NO gas propulsion	^62^
		v: N/R			
		b: *S. aureus/E. coli*			
		DL%/EE%: 6.8%/97.6%			
	Enzymatic-based UCNP and mSiO_2_ (UUmSC)	s: ≈250 nm	Urea (100 mM)	Self-diffusiophoresis	^63^
		d: 8.22 μm^2^ s^–1^			
		b: 4T1, MCF-7 cells			
		DL%/EE%: N/R			
	Soft mesoporous organosilica-based (SCC)	s: ≈250 nm	H_2_O_2_ (endogenous or 20 mM)	Self-diffusiophoresis	^64^
		d: 3.87 μm^2^ s^–1^			
		b: 143B cells			
		DL%: 8.5%			
					
Phthalocyanine (e.g., ZnPc, SiPc, etc.)	Polymeric-based IONPs (ISP-NMs)	s: 342.9 nm	H_2_O_2_ (0.2 mM)	Self-diffusiophoresis	^65^
		v: 1.66 μm s^–1^			
		b: HeLa and 4T1 cells			
		DL%: 7.2%			
	Polymeric-based (PCZN)	s: 149.4 nm	Mn(CO)_5_Br-generated CO gas	Bubble propulsion	^66^
		d: 3.04 μm^2^ s^–1^			
		b: N/R			
		DL%: 18.4%			
	mSiO_2_-based PL (mPL-Pc@PDA)	s: ≈ 200 nm;	Light	Self-thermophoresis	^47^
		v: 7.53 μm s^–1^			
		b: H22 and NIH-3T3 cells			
		DL%/EE%: N/R			
					
Methylene Blue (MB)	Magnetic mSiO_2_/Pt (J-Pt@MMS)	s: ≈ 150 nm	H_2_O_2_ (endogenous)	Bubble propulsion	^67^
		v: N/R			
		b: CCRF-CEM cells			
		DL%: 0.24%			
					
Curcumin (Cur)	Algae-based (Cur-NPs-C)	s: ≈ 2 μm	NIR light	Biological phototaxis	^68^
		v: N/R			
		b: 4T1 cells			
		DL%/EE%: N/R			
					
Black TiO_**2**_	Enzymatic-based black TiO_2_ (HTiPC)	s: 180-360 nm	H_2_O_2_ (endogenous or 0.2 mM)	Self-thermophoresis	^69^
		v: 18.71 μm s^–1^			
		b: CT26 cells			
		DL%/EE%: N/R			

a The table summarizes PS type, MNM design, motor size (s), velocity or apparent diffusion coefficient (v or d, respectively), biological model (b), drug loading or encapsulation efficiency (DL% or EE%), energy source, and propulsion mechanism. Abbreviations: N/R, not reported; RBC, red blood cells; IONPs, iron oxide nanoparticles; PL is persistent luminescent material; UCNP is upconverting nanoparticles; HSA is human serum albumin; FA, folic acid; and ZIF, zeolitic imidazolate frameworks.

As summarized in Table 1, the propulsion strategies most frequently employed in MNM-assisted PDT are light-driven and enzyme-powered mechanisms. Light-responsive MNMs offer remote and spatiotemporal control over propulsion and therapeutic activation, although their applicability in deep tissues remains constrained by a limited light penetration. NIR-responsive systems partially address this limitation because NIR light penetrates biological tissue more effectively than visible or UV light, but access to deep-seated lesions remains challenging. Conversely, enzymatic propulsion systems can take advantage of endogenous substrates such as urea or hydrogen peroxide, enabling autonomous and sustained movement, even in the absence of external energy input. Their performance, however, depends on the substrate availability and concentration, which may vary substantially across biological environments. Direct comparisons of propulsion strategies under equivalent experimental conditions remain scarce, particularly with respect to therapeutic efficacy, biosafety, and long-term *in vivo* performance. Future research should prioritize standardized evaluation frameworks and direct comparative analyses to determine which propulsion strategies yield the most robust therapeutic outcomes. The following sections examine the principal strategies used to integrate PSs with MNM architectures and discuss how PS selection and the coupling method influence overall platform performance.

## Coupling Strategies between Photosensitizers and Micro- and Nanomotors

The main strategies for coupling PS with MNMs include physical adsorption, encapsulation, covalent linkage, and electrostatic or hydrophobic interactions (Figure 2). In this section, we discuss these approaches in detail and examine how they influence PS loading, retention, ROS generation, and motor functionality, ultimately impacting the performance of the MNM-assisted PDT systems.

**Figure 2:**
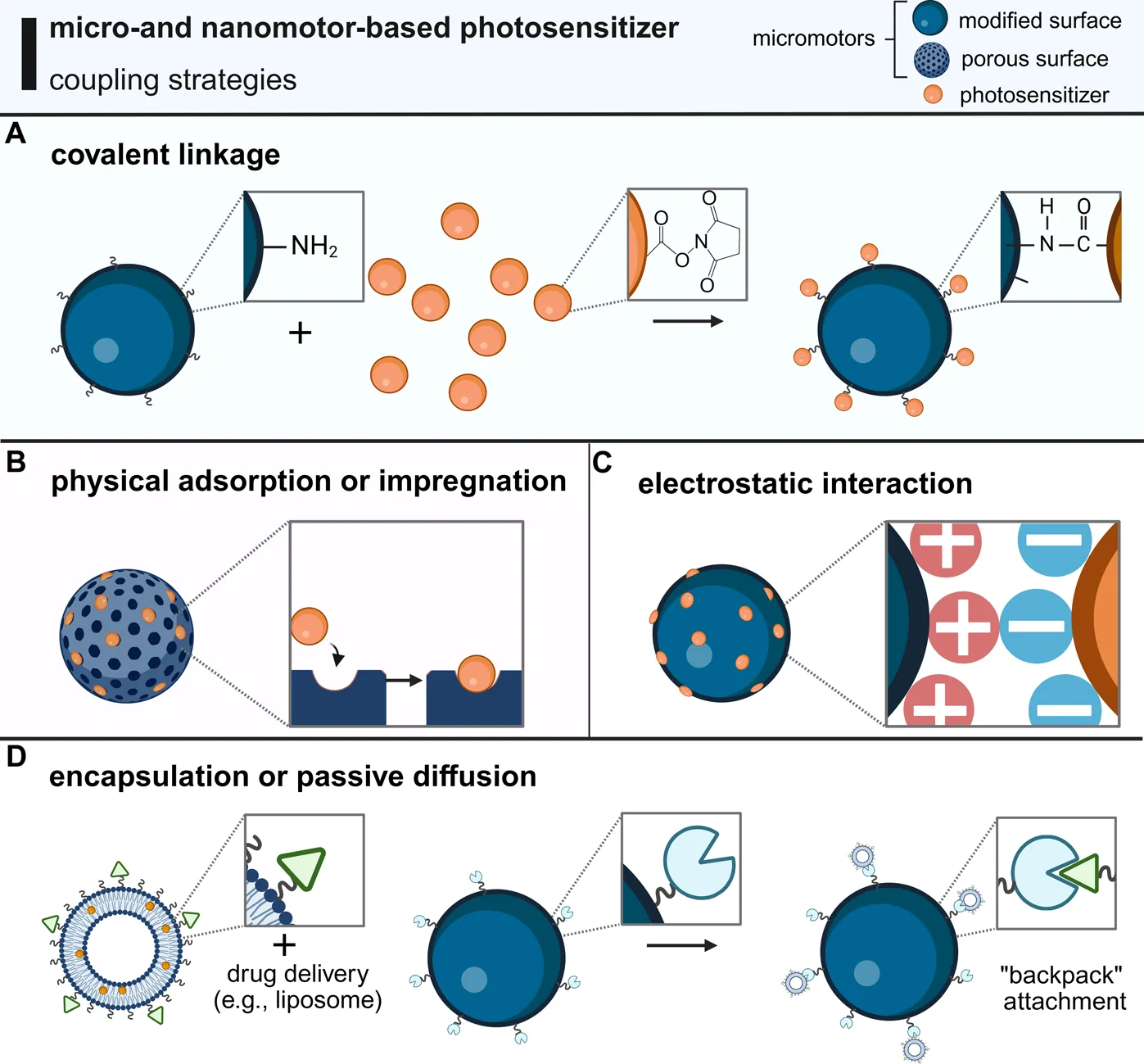
Schematic illustration of the main coupling strategies between micromotors and photosensitizers, including (A) covalent linkage, (B) physical adsorption/impregnation, (C) electrostatic interaction, and (D) encapsulation or passive diffusion. Created in BioRender. Villa, K. (2026)https://BioRender.com/k48wvhu.

### Physical Adsorption and Impregnation Methods

Physical adsorption and impregnation procedures are commonly used to incorporate PSs into MNMs containing porous, hollow, or high-surface-area architectures, particularly mesoporous silica-based materials (Figure 2).^48,70^ The surface porosity of these materials plays a crucial role, enabling the confinement of PS through hydrophobic forces, thus keeping it in a monomeric state. Numerous studies have demonstrated the successful loading of different PSs into various MNMs, including gold-based photothermal nanomotors, such as HANM@FI^58^ and Au@ZnO@SiO_2_-ICG,^48^ catalytic Janus nanomotors, including IR820@CuS/Pt^43^ and Au_2_Pt@PMO@ICG,^46^ and other hybrid catalytic or light-responsive systems based on metallic or metal-oxide cores (e.g., C&D@Z^62^ and UUmSC^63^). The loading procedure is generally performed by incubating the PS with the MNM, often in the dark, followed by repeated washing to remove unbound PS and isolating the resulting MNM-PS. PS loading is typically quantified indirectly by measuring the concentration of the unbound PS remaining in the supernatant using UV–Vis absorption or fluorescence spectroscopy and comparing it with a calibration curve. Reported values include a loading efficiency of 47.69% for HANM@FI,^58^ and encapsulation efficiency of 41.2% for DIMs.^49^ Successful incorporation is further supported by changes in zeta potential, FT-IR spectra, or changes in the characteristic optical properties of the PS, including its absorption and fluorescence signatures.^43,63^

Overall, physical adsorption and impregnation provide simple and versatile routes for integrating PSs into MNMs without requiring a covalent modification. Their main limitations are the potential for premature PS release, incomplete control over loading location, and a strong dependence of loading capacity and retention on the carrier architecture, surface chemistry, and PS properties.

### Encapsulation or Passive Diffusion Method

The encapsulation or passive diffusion method loads drugs by allowing them to diffuse into pre-formed soft-matter self-assembled structures, making it particularly suitable for polymeric colloidal motors, stomatocytes, and liposome-based systems (Figure 2).^65,66,68^ A prominent example is the light-driven biohybrid R-Motor, which uses *Chlamydomonas reinhardtii* as the propulsion unit to transport the protoporphyrin IX precursor 5-aminolevulinic acid (ALA) in liposomal backpacks.^16^ The liposomal component was prepared using the classical membrane-hydration method, achieving an ALA encapsulation efficiency of 87.8%. The liposomes were then anchored to the microorganism via host-guest interactions between ε-cyclodextrin-modified algae and adamantane-functionalized liposomes. Upon light activation, R-Motor exhibited phototactic navigation, effectively delivered ALA, and generated oxygen photosynthetically, enhancing both *in vitro* and *in vivo* PDT outcomes.^16^ A similar approach was reported for self-assembled human serum albumin (HSA) CurNPs-C MNMs, in which the natural PS curcumin (Cur) was encapsulated within HSA to form CurNPs and subsequently attached to phototactic Chlorella (C) via surface functionalization. In contrast to systems in which PSs are directly embedded within the MNM structure, for example through physical adsorption, these two examples feature PS-containing “backpacks” that function as separate formulations and do not directly contribute to MNM propulsion. Their main role is to enable active transport and controlled delivery of the phototactic cargo, thereby overcoming limitations associated with passive delivery mechanisms such as the EPR effect.^9,33^ Alternatively, PS compounds can be encapsulated directly within the MNM architecture by passive diffusion or *in situ* loading during the particle assembly, as demonstrated in PCM, stomatocyte-based ISP-NMs, GC6@cPt nanomotors and RBC-shaped magnetic hemoglobin cores.^42,54,56,65^ Notably, in the PCM system, integrating tetra­(p-hydroxyphenyl)­porphyrin (THPP) into the asymmetric thiophene-containing semiconducting polymer enabled direct photodynamic particle self-propulsion, in contrast to the pure Brownian motion observed in the dark.^42^ Similarly, GC6@cPt nanomotor was constructed by co-encapsulating chlorin e6 (Ce6), glucose oxidase (GOx), and catalase (CAT) within a cisplatin prodrug-skeletal zeolitic imidazolate framework. Beyond serving as a delivery platform for this PS, the nanomotor exploits GOx/CAT cascade catalysis to generate oxygen *in situ*, enabling autonomous propulsion and enhancing combined PDT and CTx activity.^54^ Overall, soft MNMs represent a promising next generation of drug-delivery platforms for MNM-assisted PDT. By combining efficient drug delivery, intrinsic motility, and high biocompatibility, these systems offer significant potential for enhanced photodynamic therapeutic performance.

### Covalent Linkage Method

Covalent coupling provides a robust strategy for integrating PSs with MNMs by forming stable chemical bonds between the PS and the motor scaffold (Figure 2). Common approaches include amide bond formation between amino and carboxyl groups, condensation reactions, and thiol-maleimide click chemistry. This approach has been applied to a variety of PSs, including 5,10,15,20-tetrakis­(4-aminophenyl)­porphyrin (TAPP), Ce6, and silicon phthalocyanine (SiPc).^47,61,64^

One representative example is the immobilization of TAPP onto UMSA3, comprising an upconverting nanoparticle (UCNP) core coated with mSiO_2_ and asymmetrically modified with a gold layer bearing 3-mercaptophenylboronic acid, via glutaraldehyde (GA)-mediated conjugation. GA reacts with the amino groups of TAPP to form Schiff base linkages, yielding UMSTA3; subsequent catalase functionalization produces the final UMSTCA3 nanomotor. The resulting multifunctional MNM enables a range of phototheranostic applications, such as surface-enhanced Raman scattering (SERS) sensing, fluorescence imaging, photoacoustic imaging (PAI), PDT, and PTT.^55^ Covalent attachment was also employed in the fabrication of mPL-Pc@PDA MNMs, where SiPc was conjugated to mPL-NH_2_ by a condensation reaction between its amino groups and the chlorine atoms of the SiPc.^47^

Compared with physical adsorption, covalent attachment generally provides greater PS stability and minimizes premature release during the operation. While some studies have noted that integrating PS within the MNM structure may boost propulsion through sustained radical generation and photothermal effects, this coupling approach remains relatively underexplored.^47^ Overall, this strategy offers important advantages for applications requiring prolonged stability, controlled PS localization, and multifunctional therapeutic platforms.

### Electrostatic Method

The final strategy for coupling PS compounds into MNMs uses an electrostatic interaction method. Similar to physical adsorption, this approach generally involves incubating presynthesized MNMs with the PS under mild conditions, allowing association through electrostatic attraction. Depending on the system, additional non-covalent interactions, such as hydrophobic forces or metal coordination, may further stabilize the PS within the MNM architecture.

To date, the only literature reporting MNM-assisted PDT using this coupling method is the fabrication of PDA–PEG-ICG-Fe^3+^. In this system, the PS remains associated with the nanomaterials through a combination of electrostatic and hydrophobic interactions, as well as chelating with ferric ions (Fe^3+^).^44^ ICG serves both as the PS and as the photothermal component, converting NIR irradiation into localized heating that drives thermophoretic propulsion while simultaneously enabling phototherapy.

Although electrostatic coupling offers a simple and mild loading strategy that avoids chemical modification of either the PS or the MNM, the non-covalent nature of these interactions may result in lower loading stability and greater susceptibility to PS release under physiological conditions.

## Photosensitizer for MNM-Assisted PDT

MNM-assisted PDT systems incorporate both established PSs and emerging photoactive compounds. Interest in diverse photoactive molecules has increased because of their tunable photophysical properties and potential for improved therapeutic outcomes.^16,48,67^ These primarily include porphyrin-based systems, as well as phthalocyanines, chlorins, and cyanines.^59,62,66^ Notably, other photoactive molecules not traditionally classified as PS (e.g., aggregation-induced emission luminogens (AIEgens)) have also been reported in MNM-assisted PDT.^71,72^ In parallel, biohybrid systems that incorporate intrinsic photoactive components, including chloroplasts from chlorophyll-based systems, are being explored due to their ability to enable light-driven motion via phototaxis.^68^ The following section provides a comprehensive overview of these emerging and established photoactivated compounds used alongside PDT and MNMs.

### MNM-Based Porphyrin Systems

Porphyrin derivatives are pigments widely found in nature and play essential biological roles, including light-harvesting, oxygen transport, and catalysis.^73,74^ Their unique π-conjugated structures govern their distinctive photophysical, photochemical, and electrochemical behaviors. In living organisms, porphyrins interact with proteins such as hemoglobin, where the heme group binds iron to mediate oxygen transport, and serve as chromophores in photosynthetic light-harvesting complexes.^73,74^ Beyond their natural roles, porphyrins can coordinate with a wide range of metal ions and act as versatile building blocks for supramolecular assemblies, making them highly valuable for the design of bioactive materials. Among the porphyrin derivatives incorporated into MNMs, TAPP, and THPP are representative examples (Figure 3A). Their amino or hydroxyl substituents facilitate covalent conjugation, coordination, and incorporation into porous or supramolecular structures.^54,74^ However, unlike Verteporfin, TAPP and THPP are not FDA-approved.^75,76^

**Figure 3:**
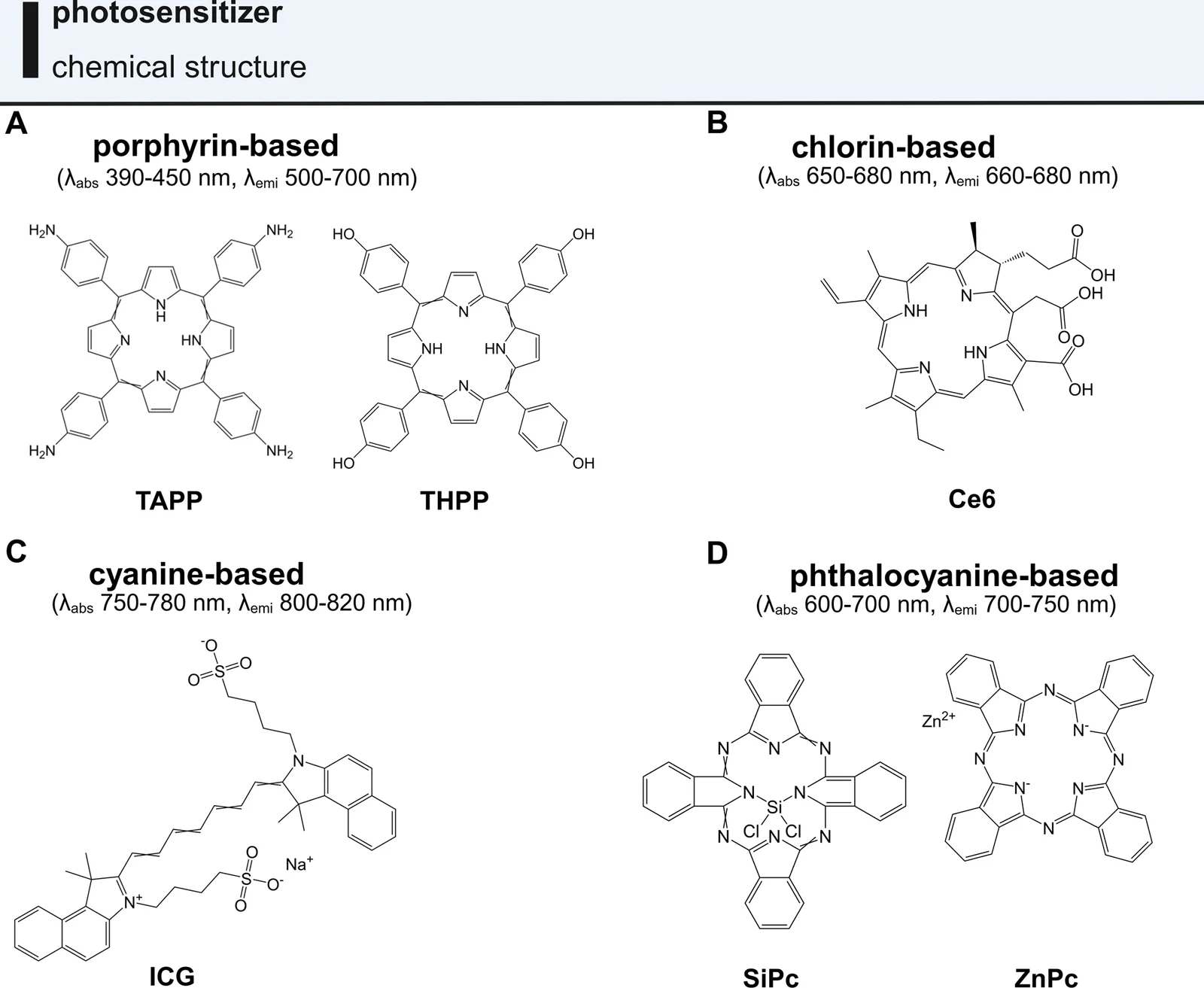
Chemical structures and characteristic absorption and emission ranges of representative photosensitizers used in self-propelled MNMs: (A) porphyrin-based PSs, (B) chlorin-based PSs, (C) cyanine-based PSs, and (D) phthalocyanine-based PSs. The indicated wavelength ranges are representative values and may vary with molecular substitution, solvent, aggregation state, and local environment.

An early example of porphyrin-based MNM-assisted PDT featured the covalent attachment of TAPP to Janus enzymatic magnetic hollow mesoporous platforms (MHSTU).^53^ Confinement and surface attachment reduced TAPP aggregation under the tested conditions and preserved its ability to generate ROS, including ^1^O_2_. Results of Φ_Δ_ show increases of over 20% compared to passive (non-motile) delivery approaches. Notably, the autonomous propulsion of MHSTU motors significantly enhances PDT efficacy by improving TAPP’s interaction with oxygen-rich microenvironments.^53^

THPP has also been explored because its peripheral substituents can be modified to tune its photophysical properties and facilitate integration with hybrid materials.^77,78^ Upon appropriate visible light excitation, THPP can promote electron-transfer processes and contribute to tumor-cell inhibition. Porphyrins have also been co-encapsulated with chemotherapeutic agents such as doxorubicin (DOX) to enable multimodal therapeutic strategies.^17,49,57,79^ The MNM-based porphyrin studies summarized in Table 2 employ different irradiation protocols. Beyond these dosimetric considerations, integrating porphyrin derivatives with MNMs offers substantial advantages, including straightforward coupling strategies and convenient spectroscopic characterizations.^73,74,78,80^ The central macrocycle of porphyrins provides versatile metal-ion coordination sites, while peripheral groups enable additional interactions such as hydrogen bonding and electrostatic interactions. Furthermore, the synergy extends to combinations with metal-based MNMs, such as Cu_2_O, allowing for in-depth exploration of electron-transfer mechanisms.^80^ Altogether, these features foster strong synergistic effects and significantly enhance the functional potential of MNM-based porphyrin hybrid systems across a range of applications beyond that of PDT.

**Table 2:** MNM-Assisted PDT Systems Employing Calculated Fluence Values (≤200 J cm^–2^)­^a^

MNM-assisted PDT system	λ (nm)	Irradiance (mW cm^‑2^)	Irradiation time (min)	Fluence (J cm^‑2^)	Ref.
UMSTCA3	808	1.5	40	3.6	^55^
UTZCG	980	1.5	40	3.6	^54^
MHSTU	450	14.2	15	12.8	^53^
R-Motor	680	40	30	72	^16^
J-Pt@MMS	635	45	30	81	^67^
PCM	405	300	5	90	^42^
GC6@cPt ZIF	660	200	10	120	^15^
SCC	660	1000	2	120	^64^
C&D@Z	660	500	5	150	^62^
PCZN	808	300	10	180	^66^
CSIL	808	1000	3	180	^60^

a MNMs abbreviations: UMSTCA3 comprises a UCNP core surrounded by an mSiO_2_ shell bearing TAPP and catalase and asymmetrically coated with an Au layer functionalized with 3-MPBA; UTZCG comprises UCNPs and TAPP incorporated into a ZIF-8 metal–organic framework (MOF) functionalized with GOx and catalase; MHSTU comprises TAPP-loaded magnetic hollow mSiO_2_ functionalized with urease; R-Motor comprises ALA-loaded liposomes anchored to the surface of C. reinhardtii by host-guest complexation; J-Pt@MMS comprises MB-loaded magnetic mSiO_2_ decorated with Pt; PCM is a THPP-loaded asymmetrical polymer colloidal motor; GC6@cPt ZIF comprises GOx, catalase and Ce6 co-encapsulated within cisplatin-skeletal ZIFs; SCC is a soft mesoporous organosilica nanomotor loaded with Ce6 and surface-functionalized with CAT; C&D@Z is a ZIF-based platform containing arginine-terminal butterfly-shaped peptide dendrimers loaded with Ce6; PCZN comprises ZnPc-loaded PDA nanobowls; and CSIL is a spinous yolk–shell C/SiO_2_@C nanomotor co-loaded with ICG and lysostaphin.

### MNM-Based Chlorin Systems

Ce6 is a partially hydrogenated derivative of porphyrins belonging to the chlorin class (Figure 3B).^61^ It is chemically known as (17S,18S)-18-(2-carboxyethyl)-20-(carboxymethyl)-12-ethenyl-7-ethyl-3,8,13,17-tetramethyl-17,18,22,23-tetrahydroporphyrin-2-carboxylic acid and is composed of four pyrrole rings, which are part of the broader tetrapyrrole family.^61^ Compared with porphyrins, chlorins contain one partially hydrogenated pyrrole ring, corresponding to reduction of one macrocyclic double bond. This structural difference enhances absorption in the red region, typically around 660-670 nm. Derived primarily from chlorophyll *a*, Ce6 retains the characteristic macrocyclic scaffold of tetrapyrroles. Still, this PS exhibits superior photosensitizing efficiency, making it widely used in PDT due to its strong light absorption and high Φ_Δ_ generation capacity, excellent biocompatibility, and proven photodynamic activity against a broad range of cancer cell lines.^81,82^

MNM-based chlorins are often engineered for combination therapies including PAI, PTT, and CTx.^62–64^ For instance, Janus mesoporous platinum-organosilica nanoparticles (PPIC nanomotors, Table 1), which incorporate Ce6 and ICG, represent a multifunctional MNM-assisted PDT platform.^61^ In this system, the platinum component catalyzes hydrogen peroxide decomposition within the tumor, enhancing both PDT and PAI functionalities, while the active motion improves tissue penetration.^61^

Several MNM-based chlorin systems have been evaluated using fluences of ≤ 200 J cm^–2^. Recently, however, UUmSC, an enzyme-driven Janus nanomotor comprising a UCNP core, an asymmetric mSiO_2_ shell loaded with Ce6, and an urease-functionalized surface, demonstrated promising *in vitro* performance at 600 J cm^–2^. The UCNP core converts 980 nm NIR irradiation into visible emission that activates Ce6, enabling PDT under this irradiation protocol.^63^ However, further *in vivo* studies are required to validate the efficacy of MNM-based chlorins using this irradiation protocol. Finally, the development of biohybrid systems primarily composed of microorganisms, such as algae (e.g., *Chlamydomonas reinhardtii* and *Euglena gracilis*), has expanded the integration of MNM-based chlorins.^16,39,83^ These motors naturally exhibit photodynamic activity through their endogenous PS (e.g., chlorophyll in chloroplasts), enabling both light-driven photosynthesis and navigation via flagellar beating controlled by light stimuli. Such MNMs provide multiple advantages, including multitasking capabilities within constrained microenvironments, precise drug delivery, and effective PDT treatment, all with high levels of controllability and adaptability. Their superior biocompatibility and inherent light-driven propulsion eliminate the need for additional synthetic or magnetic materials for navigation, making them a promising alternative among MNM-based chlorins.^16,39^

### MNM-Based Cyanine Systems

Numerous studies have explored integrating diverse photoactive compounds with MNMs to improve phototherapeutic efficacy.^43,46,49^ Among these, ICG stands out as a leading candidate due to its FDA-approved clinical use as a fluorescent diagnostic agent for ophthalmic angiography and hepatic function assessment. Its strong NIR absorption in the 700–800 nm range enables *in vivo* imaging, efficient photothermal conversion, and ROS generation, making it particularly attractive for MNM-based platforms that simultaneously integrate PDT, PTT, and light-driven propulsion (Figure 3).^84–87^ One of the first notable developments in MNM-based ICG systems involved biofriendly red blood cell-mimicking micromotors (RBCMs) designed for efficient oxygen delivery and improved PDT outcomes.^56^ These RBCMs encapsulate Fe_3_O_4_ nanoparticles, enabling acoustic propulsion and magnetic guidance toward the tumor site, while hemoglobin provides an intrinsic oxygen-carrying capacity. This combination of controlled locomotion and oxygen delivery enhances PDT efficacy by increasing local oxygen availability and amplifying light-induced cytotoxicity.^56^ ICG has also been integrated into a HSA-based, urease-powered nanomotor for combined PTT and PDT, targeting both normal (HL-7702) and cancerous (HepG2) hepatic cells.^58^ The engineered HSA-AuNR@FA@Ur@ICG (also known as HANM@FI) is composed of HSA modified with gold nanorods (AuNRs), loaded with folic acid (FA) and ICG, and coated with Ur. In this system configuration, HSA enhances the biocompatibility and reduces the cytotoxicity to normal cells, while FA enables specific targeting of HepG2 cells. ICG provides both PDT functionality and real-time fluorescence imaging, while Ur enables self-propulsion for a targeted delivery. Under NIR irradiation, this system achieves enhanced tumor ablation through a synergistic combination of PDT and PTT, further amplified by the photothermal effect of AuNRs.^58^ This targeted MNM- and ICG-guided therapy demonstrated greater tumor eradication than conventional phototherapy.

ICG was subsequently incorporated into the urease-powered bowl-shaped MPDA@ICG@Ur@Man nanomotor. The heat generated by this PS further enhances the enzyme activity and accelerates the urea decomposition. This improved motion translates into more effective phototherapy-based PDT and PTT antibacterial outcomes against *E. coli* biofilms, offering an antibiotic-free antibacterial strategy.^45^ Other approaches have integrated ICG into MNMs without relying on enzymatic propulsion.^44,46^ For example, dual-source-driven, parachute-like Janus Au_2_Pt@PMO@ICG micromotors (APIJNS) and walnut-shaped PDA–PEG-ICG-Fe^3+^ micromotors, composed of polydopamine (PDA), methoxypolyethylene glycol (mPEG-NH_2_), ICG, and chelated ferric ions (Fe^3+^), represent two non-enzymatic MNM-based cyanine platforms.^44,46^ For instance, in APIJNS, the Au_2_Pt component catalyzes H_2_O_2_ decomposition and contributes to oxygen-generating propulsion, whereas NIR-induced photothermal gradients provide an additional driving force. Reported *in vivo* studies indicate that MNM-based ICG systems can combine PTT and PDT under a range of irradiation conditions, which supports their potential for cancer therapy. More broadly, the use of ICG in the design of NIR-driven MNMs, often in combination with PDT, PTT, and/or CTx, has shown notable efficacy in eradicating pathogens, disrupting biofilms, promoting wound healing, and treating cancer.^45,59,61^

### MNM-Based Phthalocyanine Systems

Phthalocyanines (Pcs, Figure 3) are second-generation PSs that have attracted considerable interest in MNM-assisted PDT.^47,65,66^ Structurally related to porphyrins, these synthetic dyes comprise four isoindole groups, each featuring a benzene ring fused to pyrrole units and interconnected by nitrogen atoms. Pcs are recognized for their outstanding photophysical and photochemical properties, notably their high molar absorption coefficient within the therapeutic window and negligible absorption in the 400-550 nm range. This property may help to reduce the risk of skin photosensitivity following treatment. Moreover, the chemical versatility of Pcs enables their pharmacokinetic and biodistribution to be tuned through structural modification and formulation.^47,65,66^ Although various types of Pcs have been reported in the literature, zinc- and silicon-centered Pcs are the most commonly used in combination with MNMs.^47,65,66,88^ Phthalocyanine-based MNMs can combine PDT with other therapeutic modalities, such as PTT.^47^ A representative example is the PCZN nanomotor, which consists of polydopamine (PDA) nanobowls loaded with ZnPc and manganese pentacarbonyl bromide (Mn­(CO)_5_Br, abbreviated as COMn). In the presence of H_2_O_2_, COMn decomposes to release CO and form Mn^2+^. The resulting Mn^2+^ catalyzes the decomposition of endogenous H_2_O_2_ to generate O_2_, producing dual-gas propulsion driven by CO and O_2_. This design combines active transport of the ZnPc-loaded MNM with local oxygen generation, improving the overall PDT performance.^66^

### Emerging Photoactive Molecules for MNM-Assisted PDT

Beyond conventional PSs, a growing number of photoactive molecules and semiconducting materials have been incorporated into MNM platforms to expand the capabilities of PDT.^11,89,90^ Among these, organic lipophilic compounds are especially valuable in PDT because their affinity for cellular membranes enhances uptake and enables deeper penetration into tumor tissues.^4^ However, highly hydrophobic molecules tend to self-assemble in aqueous environments, leading to ACQ. This self-aggregation not only decreases solubility in pharmaceutical formulations and the bloodstream but also shortens the lifetimes of their excited triplet states. As a result, oxygen-based radical generation is diminished, compromising the overall efficacy of the PDT. In contrast, AIEgens exhibit unique behavior: they become highly emissive and show significantly enhanced ROS production upon aggregation, thus overcoming the limitations of ACQ.^83^ Although only a limited number of molecular AIEgen structures have been reported to date, their distinct properties make them especially promising for PDT and theranostic applications, as well as for integration into MNMs.^83^

Several MNM platforms have exploited these properties.^91,92^ For example, Shao et al. developed self-assembled soft-MNMs using amphiphilic block copolymers of poly­(ethylene glycol) (PEG) and AIEgenic poly­(trimethylene carbonate) (PTMC) moieties, creating theranostic AIE-stomatocytes with improved intracellular delivery and cancer cell killing.^91^ Similarly, asymmetric peanut-shaped AIEgen-functionalized polymersomes, as well as AIEgen-functionalized polymersomes with asymmetric Au nanoshells, enabled real-time tracking and NIR-triggered propulsion.^71^ This multifunctional design enhanced PDT efficacy and precision by integrating fluorescence imaging, autonomous motion, and efficient ROS generation for simultaneous therapy and visualization.^71,92^ More recently, the combination of *Escherichia coli* Nissle 1917 (EcN) and the AIEgen compound INX-2 was explored to construct biohybrid bionic micromotors (EcN@INX-2) designed to enhance tumor targeting, penetration, and retention.^72^ EcN@INX-2 exhibits NIR-II fluorescence, in addition to PDT and PTT therapeutic effects, enabling *in vivo* multimodal imaging and therapy in colon cancer models using female mice, effectively integrating MNMs with theranostic capabilities.^72^

Photoactive semiconductors have also emerged as complementary ROS-generating components in MNM-assisted PDT.^69^ TiO_2_ is a benchmark photocatalytic material with high stability and biocompatibility that can generate cytotoxic ROS through photoinduced redox reactions. A representative example is the HTiPC Janus micromotor, consisting of an NIR light-responsive black TiO_2_ nanosphere and an enzyme-functionalized periodic mesoporous organosilica (PMO) nanorod that encapsulates the TiO_2_ core. In the tumor microenvironment, the elevated hydrogen peroxide concentration serves as a fuel source, which is decomposed by CAT within the PMO domain to propel the system under irradiation.^69^ By finely tuning the TiO_2_:PMO ratio, this MNM exhibits optimized self-propulsion, enhancing both the directionality and velocity, thereby promoting cellular uptake and deep tumor penetration. Furthermore, continuous catalytic decomposition of endogenous hydrogen peroxide generates molecular oxygen *in situ*, amplifying PDT efficacy and significant tumor growth inhibition.^69^ As highlighted, many photoactive compounds exhibit synergistic effects when combined with MNMs, thereby enhancing PDT efficacy and other clinical modalities, such as CTx and PTT. Recognizing the importance of these strategies can inspire researchers to explore innovative solutions. The following section will provide a detailed overview of these approaches, light source, drug loading, effectiveness and efficiency, as well as the challenges associated with them.

## Influence of Light Irradiation on MNM-Assisted PDT

The therapeutic performance of PDT is strongly influenced by light delivery, which determines the spatial distribution of excitation and, consequently, ROS generation within the target tissue. Light propagation is governed primarily by tissue absorption and scattering, both of which depend on the wavelength of the incident light and the presence of endogenous and exogenous chromophores (e.g., hemoglobin, myoglobin, and cytochromes).^12,93^ Consequently, red-to-near-infrared (NIR) light (600–850 nm) is generally preferred for offering improved tissue penetration and enhanced performance in optical imaging and phototherapy. This spectral window is also advantageous for activating propulsion and guiding various MNMs, with light responsiveness typically reported across the 400-900 nm range. In the context of MNM-assisted PDT, the proper selection of light wavelength enables precise control over cargo delivery to target tissues, thereby improving localization and controlled activation.^94–96^

Besides wavelength, PDT efficacy depends critically on the irradiation protocol, including irradiance, irradiation time, and fluence (i.e., cumulative light dose). Irradiance refers to the radiant power delivered per unit area, whereas fluence represents the cumulative radiant energy delivered per unit area during the treatment. These parameters collectively influence ROS generation, oxygen consumption, photobleaching, and the balance between the apoptotic and necrotic cell death pathways. Importantly, identical fluence values can be achieved using different combinations of irradiance and irradiation time, which may result in distinct biological responses because oxygen depletion and thermal effects depend strongly on the irradiance.

Tables 2 and 3 summarize the irradiation parameters reported for MNM-assisted PDT systems, including wavelength, irradiance, irradiation time and calculated fluence. For clarity, the reported studies were grouped according to their calculated fluence (≤200 J cm^–2^ and >200 J cm^–2^). This grouping is intended solely to facilitate comparison of the literature and should not be interpreted as a universal dosimetric threshold or clinical classification.

**Table 3:** MNM-Assisted PDT Systems Employing Calculated Fluence Values (>200 J cm^–2^)­^a^

MNM-assisted PDT system	λ (nm)	Irradiance (mW cm^‑2^)	Irradiation time (min)	Fluence (J cm^‑2^)	Ref.
ISP-NMs	665	400	10	240	^65^
mPL-Pc@PDA	600	800	5	240	^47^
MIL88ICG@ZIF8DOX	808	1000	5	300	^57^
IR820@CuS/Pt	808	1000	5	300	^43^
RBCM	808	350	15	315	^56^
Cur-NPs-C	660	100	60	360	^68^
DIMs	808	1500	5	450	^49^
Au@ZnO@SiO_2_-ICG	808	1500	6	540	^48^
PMV/ICG/l-Arg	808	1000	10	600	^59^
UUmSC	980	1000	10	600	^63^
APIJNS	808	1000	10	600	^46^
PPIC	660	1000	10	600	^61^
HTiPC	808	1500	10	900	^69^
HANM@FI	808	2000	9	1080	^58^
PDA–PEG-ICG-Fe^3+^	808	2000	10	1200	^44^
MPDA@ICG@Ur@Man	808	2000	15	1800	^45^

a MNMs abbreviations: ISP-NMs comprise IONPs and ZnPc co-loaded into PEG-*b*-PS stomatocytes; mPL-Pc@PDA comprises persistent-luminescence nanodots embedded into mSiO_2_, conjugated with SiPc, and coated with PDA; MIL88ICG@ZIF8DOX is a core–shell dual MOF platform comprising an ICG-loaded MIL-88 core and a DOX-loaded ZIF-8; IR820@CuS/Pt comprises hollow CuS loaded with IR820 and asymmetrically coated with Pt; RBCM is biconcave, red-blood-cell-shaped magnetic hemoglobin carrier loaded with ICG; Cur-NPs-C comprises Chlorella cells functionalized with curcumin-loaded HSA nanoparticles; DIMs comprise a mesoporous silicon nanoparticle core coated with peptide-functionalized ApSF and loaded with ICG and DOX; Au@ZnO@SiO_2_-ICG comprises an Au-seeded ZnO core, an SiO_2_ shell, and encapsulated ICG; PMV/ICG/l-Arg is microneedle patch loaded with ICG and the nitric-oxide donor l-arginine; UUmSC comprises a UCNP core, a Ce6-loaded mSiO_2_ shell, and a urease-functionalized surface; APIJNS comprises an Au_2_Pt core and a parachute-like PMO shell loaded with ICG; PPIC comprises Janus mesoporous Pt–organosilica nanoparticles covalently functionalized with ICG and Ce6; HTiPC comprises a black TiO_2_ nanosphere coupled to an enzyme-modified PMO nanorod; HANM@FI comprises HSA modified with Au nanorods and loaded with folic acid, urease and ICG; PDA–PEG-ICG-Fe^3+^ comprises walnut-shaped PDA modified with mPEG-NH_2_, loaded with ICG and chelated with Fe^3+^; and MPDA@ICG@Ur@Man comprises bowl-shaped mesoporous PDA loaded with ICG and functionalized with urease and mannose.

Importantly, PDT outcomes are also strongly influenced by the irradiance. Lower irradiance conditions have been associated with reduced oxygen depletion, minimized microlocal hyperthermia, and favored apoptosis rather than necrosis.^40,97,98^ By minimizing oxygen consumption and sustaining ROS generation over longer irradiation periods, reduced irradiance can improve therapeutic outcomes in oxygen-limited environments.^99–101^ Strategic PS selection, particularly the use of PSs that favor efficient ROS production, enables researchers to partially address hypoxia-related limitations.

In addition to driving ROS generation, light may also regulate MNM propulsion through photothermal or photochemical mechanisms, depending on the platform design.^102,103^ Consequently, light simultaneously controls therapeutic activation and particle transport, adding an additional level of spatiotemporal control that is absent in conventional PDT. Although several MNM-assisted PDT platforms operate under distinct irradiation protocols,^58^ (Table 3) meaningful comparisons between studies require consideration of the complete irradiation parameters, including wavelength, irradiance, fluence, and exposure time.

## MNM-Assisted PDT *In Vitro* and *In Vivo* Applications

The integration of MNMs with PDT has enabled new therapeutic strategies that extend beyond the improvement of PS delivery. By actively transporting therapeutic agents, stabilizing photoactive compounds, and, in selected platforms, alleviating local oxygen depletion through oxygen-generating functionalities, MNMs have demonstrated an enhanced photodynamic performance against microorganisms and tumor cells in a wide range of *in vitro* studies. More recently, encouraging results obtained in animal models have further highlighted their potential for biomedical applications (Figure 4).

**Figure 4:**
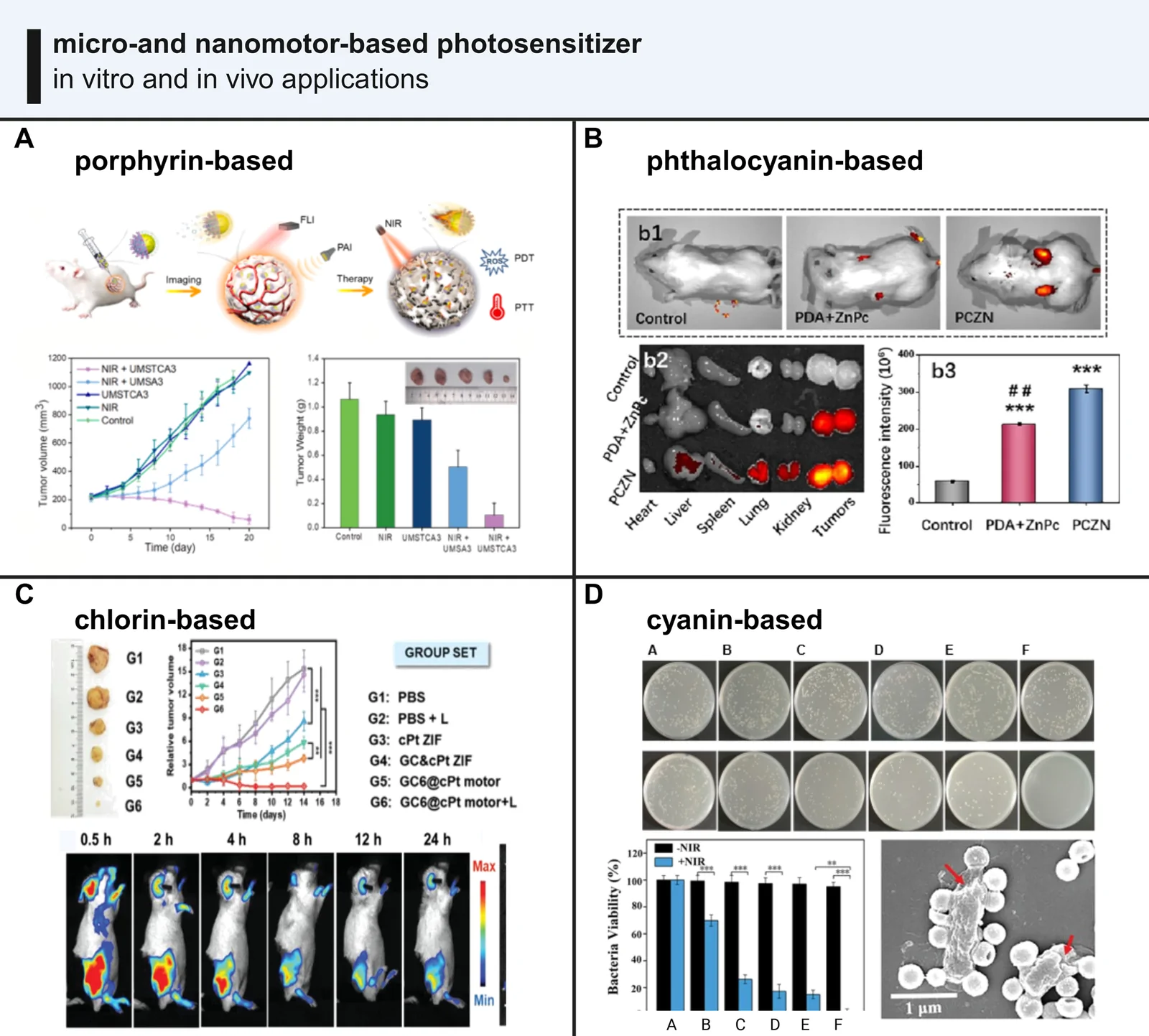
Schematic overview of micro- and nanomotor-assisted photodynamic therapy for *in vitro* and *in vivo* applications. (A) Porphyrin-based systems are represented by the UMSTCA3 nanomotor, highlighting its application for *in vivo* imaging-guided diagnosis and synergistic cancer therapy. Representative results include tumor growth profiles for the different treatment groups over 20 days and changes in the body weight of 4T1 tumor-bearing mice following treatment. Reproduced with permission from ref 55. Copyright 2022, Elsevier. (B) Phthalocyanine-based systems are represented by PCZN, showing *in vivo* fluorescence imaging, biodistribution in major organs of mice, and quantitative comparison of relative fluorescence intensities. Reproduced with permission from ref 66. Copyright 2021, Elsevier. (C) Chlorin-based systems are represented by the GC6@cPt, including photographs of excised 4T1 tumors collected on day 14, tumor growth curves, and *in vivo* bioluminescence imaging used to monitor nanomotor biodistribution at different time points. Adapted from ref 15. Copyright 2023 The Authors. Published by Wiley-VCH GmbH under the terms of the Creative Commons Attribution 4.0 International License (CC BY 4.0). (D) Cyanine-based systems are represented by MPDA@ICG@Ur@Man, showing photographs of Petri dishes, quantitative analysis of *E. coli* colony counts under different treatments, and SEM images of treated bacteria. Reproduced with permission from ref 45. Copyright 2024, John Wiley and Sons.

Despite these advances, translation to clinical use remains challenging. Long-term biodistribution, biodegradability, fuel biocompatibility, clearance, and large-scale manufacturing have been investigated only to a limited extent, while the complexity of multifunctional MNM architectures may further complicate the regulatory approval. From a clinical perspective, most applications described in this Review are still in their infancy and may be more realistically envisioned in scenarios where local administration and light accessibility are feasible, such as in superficial tumors, chronic wounds, implant-associated infections, and accessible biofilms. This section examines the interplay between reported MNM-assisted PDT and observed *in vitro* and *in vivo* photodynamic outcomes, with particular focus on the experimental parameters used, the results obtained, and the functionality and therapeutic means of these systems. To complement this discussion, Figure 4 and Table 4 summarize key *in vitro* and *in vivo* applications of MNM-based PDT. Further details on MNM-based PS and experimental parameters are provided in Tables 2 and 3.

**Table 4:** Overview of *In Vitro* and *In Vivo* Applications of MNM-Assisted PDT^a^

PS	MNM	*In Vitro* Model	*In Vivo* Model	Functionality/Therapeutic Means	Ref.
Porphyrins (e.g., TAPP)	Enzymatic-based hollow mSiO2 (MHSTU)	*E. coli*	N/A	aPDT	^53^
	Enzymatic-based MOF/UCNP (UTZCG)	HeLa cells	N/A	PDT; ST	^54^
	Enzymatic-based mSiO2, Au-coating and UCNP (UMSTCA3)	4T1 cells	4T1 bearing BALB/c mice	PDT; PTT; PAI; FLI; SERS	^55^
	Polymeric-based (PCM)	HepG2 cells	H22 bearing KM mice	PDT	^42^
					
Cyanine (e.g., ICG and IR820)	RBC-based IONPs (RBCM)	HeLa cells	N/A	PDT	^56^
	MOF-based (MIL88ICG@ZIF8DOX)	4T1 cells	4T1 bearing BALB/c mice	CTx; PTT; PDT	^57^
	ZnO/SiO_2_-based with Au seeds (Au@ZnO@SiO_2_-ICG)	*E. coli, S. aureus*, and MCF-7 cells	N/A	aPDT; PTT	^48^
	Mesoporous silicon-coated with peptides (DIMs)	4T1 cells	4T1 bearing BALB/c mice	CTx; PTT; PDT	^49^
	HSA-modified with Au nanorods and FA (HANM@FI)	HepG2 cells	N/A	PDT; PTT	^58^
	Polymeric-based (PDA–PEG-ICG-Fe^3+^)	MCF-7 cells	MCF-7 bearing BALB/c mice	PDT; PTT	^44^
	l-arginine loaded into microneedles patch (PMV/ICG/l-Arg)	*S. aureus* and HUVECs, 3T3 cells	SD rats infected with *S. aureus*	aPDT; PDT; PTT	^59^
	CuS sputtered with Pt (Janus IR820@CuS/Pt)	4T1 and NIH-3T3 cells	4T1 bearing BALB/c mice	PDT; PTT	^43^
	Enzymatic-based PDA (MPDA@ICG@Ur@Man)	*E. coli*	N/A	aPDT; PDT; PTT	^45^
	Au_2_Pt and PMO-based (APIJNS)	HeLa cells	HeLa bearing BALB/c mice	CTx; PTT; PDT	^46^
	Enzymatic-based C/SiO_2_ (CSIL)	*S. aureus* (MRSA)	C57BL/6 mice infected with *S. aureus*	aPDT	^60^
	Mesoporous Pt-organosilica (PPIC)	3T3, MCF-7 and 4T1 cells	4T1 bearing BALB/c mice	PDT; PAI	^61^
					
Chlorin (e.g., Ce6)	Enzymatic-based ZIF (GC6@cPt ZIF)	4T1 cells	4T1 bearing BALB/c mice	CTx; PDT	^15^
	ZIF-based with arginine-terminal (C&D@Z)	*E. coli* and *S. aureus*	ICR diabetic mice infected with MRSA	aPDT; DFU	^62^
	Enzymatic-based UCNP and mSiO_2_ (UUmSC)	MCF-7 and 4T1 cells	N/A	PDT	^63^
	Soft mesoporous organosilica-based (SCC)	143B cells	143B osteosarcoma-bearing mice	PDT	^64^
					
Phthalocyanine (e.g., ZnPc, SiPc)	Polymeric-based IONPs (ISP-NMs)	HeLa and 4T1 cells	4T1 bearing BALB/c mice	PDT; MRI	^65^
	Polymeric-based (PCZN)	4T1 cells	4T1 bearing BALB/c mice	PDT	^66^
	mSiO_2_-based PL (mPL-Pc@PDA)	H22 and NIH-3T3	H22 bearing Kunming mice	PDT; PTT	^47^
					
Methylene Blue (MB)	Magnetic mSiO_2_/Pt (J-Pt@MMS)	CCRF-CEM cells	N/A	PDT	^67^
					
Curcumin (Cur)	Algae-based (Cur-NPs-C)	4T1 cells	4T1 bearing BALB/c mice	PDT	^68^
					
Black TiO_2_	Enzymatic-based black TiO_2_ (HTiPC)	CT26 cells	CT26 bearing mice	PDT	^69^

a Abbreviations: N/A, not applicable; PDT, photodynamic therapy; aPDT, antimicrobial photodynamic therapy; PTT, photothermal therapy; CTx, chemotherapy; ST, starvation therapy; PAI, photoacoustic imaging; FLI, fluorescence imaging; SERS, surface-enhanced Raman scattering; MRI, magnetic resonance imaging; and DFU, diabetic foot ulcer.

Early investigations into MNM-assisted PDT, encompassing both *in vitro* and *in vivo* studies, have predominantly centered on MNM-based porphyrins. Notable examples include enzymatic-based hollow mesoporous silica (MHSTU), enzymatic-based metal-organic framework/upconversion nanoparticles (UTZCG), enzymatic-based mesoporous silica with Au-coating and UCNP (UMSTCA3), and polymeric-based constructs (PCM) (see Table 4).^42,53–55^ The enzymatically driven MHSTU was the first MNM-based PDT system to demonstrate *in vitro* photodynamic activity against bacteria (aPDT).^53^ In this study, a mixture of MHSTU, urea, and *E. coli* was exposed to blue light at an irradiance of 14.2 mW cm^–2^ (see Table 2). An extended exposure time was used to deliver the target fluence without increasing the irradiance, thereby limiting the rapid oxygen depletion and excessive local heating. Control experiments included bacteria in deionized water, urea, or MHSTU under both light-irradiation and dark conditions. Exposure to blue light in the absence of urea resulted in 39.6% bacterial death compared with dark controls. However, when the MHSTU motors were fueled with urea and subjected to aPDT treatment, the death rate significantly increased to 72.5%.^53^ Two factors may account for this enhancement: (i) nonmotile MHSTU remains confined to local oxygen-limited microenvironments, whereas urea-driven motion allows the motors to access oxygen-rich regions and increase ROS generation; and (ii) oxygen-derived ROS, particularly ^1^O_2_, have short diffusion ranges under physiological conditions, requiring close proximity between the PS-containing motors and bacterial cells. Thus, self-propulsion improves the spatial distribution of the PS and access to oxygen-rich regions, thereby enhancing ROS generation and bacterial inactivation.^53^ Later, these findings were further supported by another MNM-based porphyrin, which demonstrated notable biological photodynamic activity against HepG2 and HeLa cells (see Table 2 and Table 4).^42^ The THPP-loaded asymmetrical thiophene-containing semiconducting conjugated polymer colloidal micromotor (PCM) was subsequently evaluated *in vivo* following intravenous administration to tumor-bearing mice. Control groups received a PBS buffer, light irradiation alone, or PCM treatment without light exposure. Tumor sites were irradiated for 5 min every 2 days over a 14-day period at an irradiance of 300 mW cm^–2^, resulting in approximately 91% tumor inhibition due to the dual photothermal and photodynamic effects (see Table 2 for light treatment parameters). No significant lesions, alterations in blood parameters, or histological abnormalities were observed following treatment.^42^ To date, only PCM and UMSTCA3 have demonstrated a proof of concept for preclinical *in vivo* studies using MNM-based porphyrins. However, both porphyrin derivatives used in these MNM fabrications display complex pharmacokinetics, low selectivity, and challenging biodistribution, limiting their clinical translation and FDA approval prospects. Since PDT performed under conditions involving excessive light delivery or prolonged irradiation may accelerate oxygen consumption at the treatment site, many pioneering porphyrin-based studies have employed controlled irradiation regimes (Table 2). Furthermore, as previously highlighted, classical porphyrins primarily absorb light at wavelengths that limit tissue penetration, which restricts their application to superficial tumors. To address these challenges and enhance PDT bioapplications, various strategies have been explored, including the synthesis of new PS and AIEgens, the integration of UCNP and enzymatic components into MNM-assisted PDT, and different coupling strategies between PS and MNMs.^55,63,104^ Subsequent research revealed that integrating PDT with MNM-based systems results in a pronounced synergistic effect, improving treatment outcomes.^37,39,57^ MNM-based cyanine platforms can enhance PDT through combined ROS generation and photothermal effects.^45,48,58^ In some systems, photothermal conversion also contributes to self-propulsion. For example, Janus IR820@CuS/Pt displays catalytic and photothermal propulsion driven by its Pt and IR820 components, whereas HANM@FI combines enzymatic propulsion with photothermal functionality.^43,58^ Under the irradiation conditions summarized in Table 3, both systems showed longer trajectories under combined stimulation than under either stimulus alone (NIR/H_2_O_2_ for IR820@CuS/Pt and NIR/urea for HANM@FI). Similarly, Au@ZnO@SiO_2_-ICG exhibited increased mean squared displacement following ICG incorporation under NIR irradiation, consistent with enhanced self-thermophoretic propulsion.^48^ To advance a multimodal strategy combining PDT, PTT, and CTx, Wu et al. introduced the MOF-based MIL88ICG@ZIF8DOX system (see Table 3 and Table 4).^57^ This MNM coencapsulates ICG and DOX within a nanoporous MIL-88 core and ZIF-8 shell, enabling precise *in situ* delivery of chemotherapeutics, while efficiently converting endogenous hydrogen peroxide to oxygen, enhancing therapeutic efficacy and alleviating local hypoxia. Both *in vitro* and *in vivo* studies demonstrated that MIL88ICG@ZIF8DOX is an effective, safe, and targeted antitumor platform even under irradiation conditions described in Table 3.^57^ Recently, several researchers have demonstrated the effectiveness and safety of MNM-assisted PDT systems across a range of irradiation protocols, consistently achieving robust *in vitro* and *in vivo* antitumor effects (see details in Table 3). While alternative compounds such as Cur and Pcs are occasionally reported, the vast majority of systems rely on MNM-based ICG.^65,68^ The favorable photophysical properties, biocompatibility, and pharmacokinetics of ICG support its widespread use in the MNM-assisted PDT. As the field advances, newly developed NIR-PSs, particularly those with a Type I PDT mechanism, appear to be promising candidates for this combination and are expected to expand the therapeutic arsenal.

Despite the improvements in therapeutic efficacy frequently being attributed to MNM propulsion, many reported platforms simultaneously incorporate oxygen-generating catalysts, oxygen carriers, photothermal agents, chemotherapeutics, or targeting ligands, making it difficult to attribute benefits solely to the motion. Systematic comparisons with matched nonmotile controls, physiologically relevant hypoxic models, three-dimensional (3D) tumor spheroids, and clinically representative biofilm models remain scarce. In addition, most studies to date have been conducted in simplified *in vitro* systems or small animal models, often relying on exogenous fuels or experimental conditions that do not fully mimic the physiological tumor microenvironment. Many of those systems require exogenous fuels, whose clinical availability, toxicity, and safe concentrations remain uncertain. Likewise, enzyme-driven MNMs may face challenges related to enzyme stability, immunogenicity, and the potential loss of catalytic activity under physiological conditions. The long-term fate of inorganic components also remains insufficiently investigated, particularly regarding degradation, dose tolerance, accumulation, and clearance from the body. Therefore, these challenges must be addressed through comprehensive studies before MNM-assisted PDT can advance toward clinical translation. Future preclinical evaluation incorporating these factors will be critical for accurately defining the therapeutic contribution of MNM propulsion and establishing the translational potential of the MNM-assisted PDT.

## Challenges and Future Directions

MNM-PDT has evolved rapidly from early proof-of-concept demonstrations to increasingly sophisticated therapeutic platforms. The studies reviewed here indicate that introducing active motion into PDT systems can improve PS distribution, enhance penetration into complex biological environments, and sustain photodynamic activity under conditions where passive formulations fail. Nevertheless, despite these advances, several scientific and translational challenges remain that must be addressed before MNM-PDT can mature into a broadly applicable phototherapeutic strategy.

From a materials and propulsion perspective, a wide diversity of MNM architectures have been reported, including enzyme-powered nanomotors, catalytic Janus motors, light-driven polymeric or semiconductor systems, and biohybrid micromotors based on microorganisms or cells. Among these, enzyme-driven and catalytic Janus nanomotors currently appear to be the most promising for PDT applications, as they couple autonomous propulsion with oxygen generation and perform reliably in physiologically relevant media. These systems have demonstrated enhanced tumor penetration, while hypoxia modulation has primarily been achieved through additional oxygen generating functionalities, particularly in combination with NIR irradiation. Light-driven and semiconductor-based MNMs offer unique opportunities for spatiotemporal control, although their application *in vivo* remains relatively limited because of challenges associated with light penetration, propulsion efficiency, photothermal effects, and tissue tolerance. Biohybrid MNMs, in contrast, provide excellent biocompatibility and can exploit intrinsic biological functionalities, such as taxis and photoresponsiveness. However, issues related to long-term stability, batch-to-batch reproducibility, and regulatory approval remain unresolved. Despite the growing diversity of MNM-assisted PDT platforms, a deeper understanding of the relationships among MNM composition, propulsion strategy, biodistribution, and therapeutic outcomes is still needed. Future efforts should therefore prioritize systematic comparative studies and standardized evaluation protocols to identify the design parameters that most strongly influence the PDT performance and translational potential.

Photosensitizer selection represents a second critical challenge. To date, most MNM-PDT systems rely predominantly on well-established type II photosensitizers, including porphyrins, chlorins, and phthalocyanines. NIR-responsive cyanines are particularly common in recent *in vivo* studies because they combine red-shifted absorption with fluorescence imaging and photothermal activity. In contrast, type I photosensitizers, which generate radical species and are generally less dependent on molecular oxygen than type II pathways, have not yet been systematically explored in MNM platforms. Aggregation-induced emission luminogens and inorganic photoactive materials also remain underrepresented despite their potential to mitigate aggregation-induced quenching and improve photostability. Future progress will likely require the joint design of the photosensitizer and motor architecture so that excitation wavelength, ROS-generation pathway, propulsion mechanism, and biological function are optimized as an integrated system rather than considered independently.

Another major bottleneck is the lack of consistent methods for evaluating the photodynamic performance in MNM-assisted PDT. Variations in light fluence, irradiation protocols, oxygen availability, and ROS detection methods currently limit meaningful comparisons across studies. This challenge is intensified by the dynamic nature of MNMs, where motion alters the local PS distribution, mass transport, and oxygen availability. Quantitative assays that evaluate ROS generation, oxygen modulation, and motor activity under biologically relevant conditions are, therefore, needed. In particular, the use of compositionally matched motile and nonmotile controls will be essential to distinguish the respective contributions of propulsion, oxygen-generating functionalities, and photochemistry to the observed therapeutic response.

Finally, clinical translation imposes additional constraints related to biocompatibility, clearance, manufacturing, and regulatory acceptance. Many current MNM-PDT platforms rely on complex hybrid structures or multiple functional components, which may limit the scalability and reproducibility. Additional manufacturing challenges, including sterilization without compromising the MNM integrity, propulsion performance, or photosensitizer activity, must also be addressed to enable clinical translation. Simpler architectures based on biodegradable, bioinspired, or clinically familiar materials are therefore likely to facilitate safe clearance after therapy, while reducing long-term biosafety concerns. In parallel, the integration of molecular imaging modalities, image-guided navigation, and AI-assisted control algorithms may further improve real-time tracking, targeting, and treatment personalization in future MNM-PDT systems. Additionally, validation in clinically relevant large animal models, which could better reproduce human-scale transport barriers and biodistribution profiles, has not yet been demonstrated.

Further progress in MNM-assisted PDT will require systematic identification of the micromotor architectures, photosensitizer chemistries, and irradiation regimes that provide reproducible advantages under biologically relevant conditions. In particular, correlating propulsion mechanisms with photochemical pathways and treatment parameters will be essential to defining the realistic scope and limitations of MNM-based PDT across oncological and antimicrobial applications.

## Data Availability

Data will be made available on request.
